# Acute Otitis due to *Vibrio fluvialis* after Swimming

**DOI:** 10.1155/2012/838904

**Published:** 2012-06-17

**Authors:** Ping-Jen Chen, Chi-Chou Tseng, Huan-Tee Chan, Chien-Ming Chao

**Affiliations:** ^1^Department of Surgery, Chi Mei Medical Center, 201, Taikang Village, Liouying, Township, Tainan 736, Taiwan; ^2^Department of Intensive Care Medicine, Chi Mei Medical Center, 201, Taikang Village, Liouying, Township, Tainan 736, Taiwan

## Abstract

A 40-year-old female presented with purulent exudate through the left auditive duct and pain in the left ear region, which intensified during mastication. After collection of the pus from the left ear lesion, amoxicillin-clavulanic acid for seven days was prescribed for a presumed diagnosis of acute otitis. Four days later, the pus culture grew *V. fluvialis* which is further identified by API 20E identification system (bioMérieux). Following the successful completion of a course of antibiotics, the patient recovered completely and without complication. To the best of our knowledge, this is the first case of *Vibrio fluvialis* otitis after swimming in an immunocompetent patient.

## 1. Introduction

*Vibrio* species always causes human infection through the exposure of contaminated seawater or ingestion of contaminated seafood. Acute gastroenteritis with diarrhea has been reported to be the most common clinical presentation of *V. fluvialis *infection whilst extraintestinal infections such as bacteremia, peritonitis, hemorrhagic cellulitis, and cerebritis have been rarely reported. Here, we report the first case of *Vibrio fluvialis *otitis in an immunocompetent patient after swimming.

## 2. Case Report

A 40-year-old female visited the hospital because of purulent exudate through the left auditive duct and pain in the left ear region, which intensified during mastication. She denied any accompanying symptom such as fever, chills, headache, tinnitus, hearing impairment, diarrhea, or flatulence. She did not have a history of diabetes mellitus, end-stage renal disease, liver cirrhosis, or an immunocompromised condition. Six days prior, she had been participating in swimming in a pool of seawater. Her vital signs were a body temperature of 36.4°C, pulse rate of 65/min, respiratory rate of 20/min, and blood pressure of 109/77 mmHg. Physical examination was unremarkable except for the oedematous and erythematous external auditive duct that was associated with a purulent discharges. Laboratory examination results were within normal references ranges, including her white blood cell count of 7,900/mm^3^ (54.8% neutrophils). After collection of the pus from the left ear lesion, amoxicillin-clavulanic acid for seven days was prescribed for a presumed diagnosis of acute otitis. Four days later, the pus culture grew *V. fluvialis* and *Alcaligenes xylosoxidans*.* Vibrio* isolate is oxidase positive, esculin negative, and string test positive, and *V. fluvialis *is further identified by API 20E identification system (bioMérieux). Susceptibility testing of *V. fluvialis* revealed it to be sensitive to cefazolin and amoxicillin-clavulanic acid, amikacin, ceftazidime, ciprofloxacin, cefuroxime, gentamicin, flomoxef, piperacillin/tazobactam, and cefpirome, but resistant to ampicillin. Following a successful course of antibiotics, the patient made an uneventful recovery during followup.

## 3. Discussion

*V. fluvialis*, gram-negative, curved, rod-shaped bacteria, are ubiquitous in the marine environment and are usually found in temperate or subtropical areas, such as Taiwan [1–3]. Although *V. fluvialis *was first isolated by Furniss et al. in 1977 [4], it rarely caused human infection. Acute gastroenteritis with diarrhea was the most common clinical presentation of *V. fluvialis* infection [5]. To the best of our knowledge, *V. fluvialis *has only been reported once before, but in an HIV-infected patient with CD4 count of 123/mm^3^ [6]. 

As a marine microorganism, *V. fluvialis* always causes human infection through wound exposure to seawater or ingestion of raw seafood. In our patient, the portal of entry of the organism was most likely to be the direct exposure to contaminated sea water that permitted the propagation of the organism. This suggests that the clinicians should be alerted to the possibility that *V. fluvialis *may be an important pathogens causing human infections after participating in water activities such as boating, fishing, and swimming, or any exposure to a marine environment or animals.

In consistent with a prior report [6], the condition of our patient improved after antibiotic therapy. Although the experience of managing acute otitis caused by *V. fluvialis *is limited, appropriate antibiotic agents are supposed to cure this rare pathogen-related otitis.

In conclusion, we report a case of acute otitis caused by *V. fluvialis* in an immunocompetent patient after swimming; the clinical outcome was favorable after appropriate antibiotic management. This case further expands the spectrum of infection caused by *V. fluvialis* and raises the possibility of *V. fluvialis* as one of the causes of otitis with exposure history, even in healthy subjects.
